# Correlates of Self-Estimated Intelligence

**DOI:** 10.3390/jintelligence8010006

**Published:** 2020-02-10

**Authors:** Adrian Furnham, Simmy Grover

**Affiliations:** 1 Norwegian Business School (BI), Nydalveien 37, 0484 Oslo, Norway; 2 Department of Experimental Psychology, University College London, London WC1H 0AP, UK; harsimran.grewal.13@ucl.ac.uk

**Keywords:** self-estimated, intelligence, sex differences, attitudes

## Abstract

This paper reports two studies examining correlates of self-estimated intelligence (SEI). In the first, 517 participants completed a measure of SEI as well as self-estimated emotional intelligence (SEEQ), physical attractiveness, health, and other ratings. Males rated their IQ higher (74.12 vs. 71.55) but EQ lower (68.22 vs. 71.81) than females but there were no differences in their ratings of physical health in Study 1. Correlations showed for all participants that the higher they rated their IQ, the higher their ratings of EQ, attractiveness, and health. A regression of self-estimated intelligence onto three demographic, three self-ratings and three beliefs factors accounted for 30% of the variance. Religious, educated males who did not believe in alternative medicine gave higher SEI scores. The second study partly replicated the first, with an *N* = 475. Again, males rated their IQ higher (106.88 vs. 100.71) than females, but no difference was found for EQ (103.16 vs. 103.74). Males rated both their attractiveness (54.79 vs. 49.81) and health (61.24 vs. 55.49) higher than females. An objective test-based cognitive ability and SEI were correlated *r* = 0.30. Correlations showed, as in Study 1, positive relationships between all self-ratings. A regression showed the strongest correlates of SEI were IQ, sex and positive self-ratings. Implications and limitations are noted.

## 1. Introduction

Self-estimated intelligence (SEI) is a topic of considerable current interest for various reasons (Gignac 2018; Herreen and Zajac 2018; Howard and Cogswell 2018; Keefer 2015). First, to help people with poor insight into their performance, notably people whose self-estimates are very different from their objective scores (Schlösser et al. 2013; Chan and Martinussen 2015; Zell and Krizan 2014). Second, to look at the individual differences and processes (like social desirability, hubris, and ability test experience) that lead to (in)accurate insight about abilities (Paulhus et al. 1998). Third, to assist in self-awareness as it relates to career choice (Ackerman and Wolman 2007).

This study takes the current literature forward by looking at sex, but also other individual difference correlates of self-estimated intelligence, to try to understand the processes underlying the phenomenon.

This area has received various important reviews (Freund and Kasten 2012; Syzmanowicz and Furnham 2011; von Stumm 2014) and continues to attract many papers (Heck et al. 2018; Kaufman 2012, 2019; Neto 2019). It should also be noted that this literature has been extended to the self-assessment of other features, like emotional intelligence (Petrides and Furnham 2000) and creativity (Kaufman 2019).

It should be noted that, in nearly all these studies, intelligence only refers to self-estimated intelligence, not psychometrically tested intelligence. Overall, people are reasonably accurate at estimating their intelligence score, with correlations between estimates and actual scores typically between *r* = 0.2 and *r* = 0.4 (Furnham 2001). Results seem reasonably consistent irrespective of the IQ test used.

Studies have been done cross-culturally from Austria (Stieger et al. 2010) to Spain (Perez et al. 2010), Switzerland (Proyer 2011), and Wales (Workman 2004). Recent studies continue to be done in many countries, including Pakistan (Shahzada et al. 2014), Russia (Kornilova and Novikova 2012), and Tanzania (Dixon et al. 2016). Gender and cultural differences in self-estimates of ability have also been examined (Ivcevic and Kaufman 2013). One study compared sex differences in self-estimated intelligence across 12 countries (von Stumm et al. 2009). With no exceptions, all the above studies show a significant sex difference, with males giving higher estimates than females. However, there are more dramatic differences between countries, with both sexes from African countries giving higher self-estimates than those not from African countries.

It should be pointed out that these results could be interpreted in light of Lynn’s (2017) developmental theory of actual sex differences in psychometrically assessed IQ scores. A number of studies by Lynn and colleagues (Lynn et al. 2002, 2009, 2004) have demonstrated, using different samples and tests, a five-point advantage for males. Thus, it may be suggested that the consistent finding in the sex difference literature reflects the reality of sex differences in intelligence, though there would be many who refute this view.

Some of these self-estimated studies have also investigated participants’ beliefs about intelligence (such as whether there are sex differences) and their experiences of tests to examine whether these beliefs are related to self-estimates (Furnham and Fukumoto 2008). There have also been a number of systematic studies on self-estimates of Domain Masculine Intelligence, namely Numerical, Spatial and Verbal intelligence (Storek and Furnham 2012, 2013a, 2013b, 2014, 2016), as well as reversal intelligence (Neto et al. 2016; Neto et al. 2017).

Furnham (2017) summarised findings from over 40 studies in this area: *First*, males of all ages tend to estimate their (overall) general intelligence about 5 to 15 IQ points higher than do females, usually around one standard deviation above the norm. *Second*, when judging “multiple intelligences” males estimate their spatial and mathematical (numerical) intelligence higher but emotional intelligence lower than females. *Third*, people believe these sex differences occur across the generations: throughout the generations in one’s family, males are judged more intelligent than females. *Fourth*, sex differences are cross-culturally consistent. *Fifth*, the correlation between self-estimated and test-generated IQ is positive and in the range of *r* = 0.2 to *r* = 0.5, suggesting that you cannot use self-estimated scores as proxy for actual test scores. *Sixth*, those who score high on IQ but give low self-estimates tend nearly always to be female, while those with the opposite pattern (high estimates, low scores) tend to be male. *Seventh*, most people say they do not think there are sex differences in intelligence, but those who have taken tests and received feedback seem to give higher scores.

The questions addressed in this paper’s two studies are about other belief correlates of SEI. Many studies have looked at demographic predictors, particularly sex, age, and education. Others have looked at the relationship between SEI and IQ test taking as well as beliefs about intelligence. The studies reported here look at a wider series of factors. First, they examine whether self-rated intelligence is associated with other self-ratings of desirable characteristics like attractiveness, emotional intelligence, and health, suggesting that SEI is part of a more general self-esteem factor. It also considers whether SEI is rated to other more general belief systems like a religious belief or belief in alternative medicine. The second study also explores the relationship between SEI and psychometrically assessed IQ.

## 2. Study 1

This study focused on two types of variables as they relate to SEI. The first were other self-ratings of things like attractiveness, health and EQ. The question concerns a general “feel good” factor in all self-ratings: that is, that nearly all self-ratings of physical and psychological factors are positively related. Second, we looked at beliefs about religion and alternative medicine, because various studies have shown that more intelligent people were generally more sceptical about these issues than less intelligent people. Based on the previous literature, we hypothesised that males would rate their IQ higher (H1), but EQ lower (H2), than females. This is based on the review of Furnham (2001) and the data from Petrides and Furnham (2000) We also predicted SEI would be significantly positively correlated with ratings of EQ (H3), attractiveness (H4), health (H5) and general optimism (H6) (based on the work of Ackerman and Wolman, 2007) but negatively correlated with being religious (H7) and believing in alternative medicine (H8) (which is based on Furnham, 2001 work on self-estimated intelligence and ideology).

### 2.1. Method

#### 2.1.1. Participants

Overall, there were 517 adults, of which 259 were male and 258 females. They were, on average, 29.52 years old (SD = 10.17). Just under half (44.7%) of participants reported having at least a university degree and the average years of formal education for the sample was 12.51 (SD = 5.21).

#### 2.1.2. Questionnaire

Participants were asked to estimate, on a scale from 0–100, their *intelligence* (Male = 74.12; SD = 13.47 Female = 71.55: SD = 14.18; F(1,511) = 4.43, *p* < 0.05, *d* = 0.18); *emotional intelligence* (Male = 68.22; SD = 19.72 Female= 71.81: SD = 19.10; F(1,515) = 4.43, *p* < 0.05, *d* = 0.19); *physical health* (Male = 70.14; SD = 19.39 Female = 67.10: SD = 19.60; F(1,514) = 3.13, *p* > 0.05) *physical attractiveness* (Male = 60.51; SD = 19.09 Female = 56.01: SD = 19.57; F(1,509) = 6.92, *p* < 0.01, *d* = 0.24). Using a 9-point scale from 1 = Not at all to 9 = Very, they rated to what extent they were an optimist (Mean = 5.68, SD = 1.95); how religious they were (Mean = 2.09; SD = 2.75); and whether they believed in complementary and alternative medicine (CAM) (Mean = 4.11, SD = 2.41).

#### 2.1.3. Procedure

Participants were recruited online using the Prolific platform. They were told their anonymous results would be used for analysis. They were paid £1.00 for participation. The test took, on average, 8 min to complete. Ethics committee permission was sought and received.

### 2.2. Results

First, a correlation matrix was computed for all variables (See Table 1). The majority of hypotheses were confirmed: H1, H2, H3, H4, H5 and H6. However, a significant positive correlation was found between religiousness and self-rated IQ, and no significant correlation was found between belief in CAM and self-rated IQ. The highest correlations were between the self-assessed variables: self-estimated intelligence, emotional intelligence, physical attractiveness and physical health. This indicated that those who rated their IQ highly believed they were more emotionally intelligent, attractive, healthy, optimistic and religious.

Second, a multiple stepwise regression was calculated with SEI as the predictor variable. Age, sex and education were first entered, then SEEQ, attractiveness and health; then optimism, religious and believers in CAM were entered. The regression indicated that the first step accounted for 2% of the variance (F(3,482) = 3.35, *p* < 0.05) and sex (Beta = −0.106, *t* = −2.28, *p* < 0.05) and years of education (Beta = 0.092, *t* = 2.02, *p* < 0.05) were significant. The second step added 27% variance (F6,479) = 31.93, *p* < 0.001) and the final step explained 1.5% (F9,476) = 22.68, *p* < 0.001) of the variance. The final step showed that five variables were significant predictors of SEI: SEEQ (Beta = 0.26, *t* = 5.96, p < 0.001), self-rated attractiveness (Beta = 0.27, *t* = 5.91, *p* < 0.001), self-rated health (Beta = 0.13, *t* = 3.10, *p* < 0.01), religiousness (Beta = 0.10, *t* = 2.69, *p* < 0.01) and belief in CAM (Beta = −0.09, *t* = -2.18, *p* < 0.05).

## 3. Study 2

This study attempted to replicate, but extend, the first study with a population sample. This study added two additional variables. The first was psychometrically assessed IQ. We used a simple 10 item scale which we had used before, and which had a normal distribution and evidence of construct validity (Grover 2019). We hypothesized that correlations between SEI and IQ would be positive and significant (H1). We also assessed Beliefs in Conspiracy Theories (CT). Previous work showed that those who held more religious beliefs and less belief in science tended to give themselves lower SEI, possibly because they were indeed less intelligent or else less convinced of the scientific credibility of IQ tests. However, the results of the previous study did not support this, therefore a more extensive measure will be used in the current study. Previous research had shown that science sceptics tend to believe in conspiracy theories, and hence we added this variable, which has never been used in this research area (Swami et al. 2011). We predicted that the CT score would be significantly negatively associated with both SEI (H2) and actual IQ scores (H3).

### 3.1. Method

#### 3.1.1. Participants

Overall, there were 475 adults, of which 240 were male and 235 females. They were, on average, 20.98 years old (SD = 12.32). Nearly a third (30.7%) had a high school certificate as their highest qualification, a further third (36.4%) had an undergraduate degree and nearly a fifth (18.9%) had a post-graduate degree.

#### 3.1.2. Questionnaire

Many of the questions were identical to those in Study 1. However, in this study, participants were asked to estimate their IQ (cognitive ability) and Emotional Intelligence on a normal distribution used in many previous studies (range 1–200, with means of 100, and SDs labelled from 70 to 145. As in Study 1, they also estimated on a 0–100 scale their physical attractiveness (Mean = 52.34; SD = 19.20) and physical health (Mean = 58.40; SD = 22.35). Using a 9-point scale from 1 = Not at all to 9 = Very, they rated to what extent they were an optimist (Mean = 4.52, SD = 1.57); how religious they were (Mean = 2.06; SD = 1.55); and whether they believed in complementary and alternative medicine (CAM) (Mean = 3.63, SD = 1.82).

However, two additional issues were tested: IQ and beliefs in CTs. They also completed a 10-item general knowledge intelligence test with items such as GK4. What score is obtained by hitting the bull’s eye in darts? What is the unit of sound intensity? Who wrote “Of mice and men”? The alpha of this sale was 0.89.

Finally, to explore their skepticism, they completed a measure of belief in Conspiracy Theories (BCTI; Swami et al. 2011), a 15-item measure that describes a range of internationally popular conspiracy theories. Participants rated their belief that each conspiracy was true on a 9-point scale, ranging from 1 (*Completely false*) to 9 (*Completely true*). In the present study, Cronbach’s α for the BCTI was 0.91.

Finally, out of interest, two further questions were asked. To further explore the issues of religious beliefs, participants were also asked if they believed in life after death: 42% said yes, and 58% said no. To further explore the issue of ability, they were asked how many languages they spoke competently: 80% said one, 17% two and 3% three.

#### 3.1.3. Procedure

Participants were recruited on-line using the Prolific platform. They were told their anonymous results would be used for analysis. They took, on average, 10 min to complete this test. They were paid £1.00 for this participation. Ethics committee permission was sought and received.

### 3.2. Results

First, a *t*-test was used to examine sex difference in IQ, SEI, SEEQ, SATT (Self Assessed Attractiveness), SHEA (Self Assessed Health). There was no difference in the scores (0–10) on the short cognitive ability test Males = 4.17 (SD = 1.68); Females = 4.11 (SD = 1.73) F(1,473) = 0.16, *p* > 0.05. For SEI Males = 106.88 (SD = 13.64); Females = 100.71 (SD = 14.10) F(1,473) = 23.44, *p* < 0.001 (d = 0.45). For SEEQ Males = 103.16 (SD = 12.80); Females = 103.74 (SD = 13.54) F(1,472) = 0.23, *p* > 0.05). For SATT Males = 54.79 (SD = 18.10); Females = 49.81 (SD = 20.00) F(1,471) = 8.09, *p* < 0.01 (d = 0.22). For SHEA Males = 61.24 (SD = 20.25); Females = 55.49 (SD = 24.02) F(1,473) = 7.96, *p* < 0.01 (d = 26). There was no sex difference in CT score or languages spoken. However there was a significant difference for belief in life after death Males = 1.67 (SD = 0.47); Females = 1.49 (SD = 0.50) F(1,471) = 16.48, *p* < 0.001 (d = 0.37).

Second, a correlation matrix was computed for all variables (See Table 2). The highest correlations for SEI were SEEQ, belief in CAM and conspiracy theories, ratings of health, and attractiveness. This indicated that those who tended to rate their IQ highly believed they were more emotionally intelligent, attractive, and healthy, but were not believers in CAM or conspiracy theories. The correlation between SEI and IQ was r = 0.30, which is in accordance with previous studies and confirms H1.

Table 2 also shows the correlations with IQ. The results showed that more intelligent people were less religious and less optimistic. Belief in Conspiracy Theories (CTs) correlated significantly negatively with SEI (*r* = −0.14, *p* < 0.01) and IQ (*r* = −0.14, *p* < 0.01), confirming H2 and H3. Those who did not believe in life after death gave higher SEI estimates (*r* = 0.19, *p* < 0.001) and had higher IQ scores (*r* = 0.10, *p* < 0.05). The number of languages a person spoke was positively correlated with SEI (*r* = 0.12, *p* < 0.05) but not with IQ (*r* = −0.05). To examine whether males were less accurate at estimating their IQ than females, the file was split: for males, the correlation between IQ and SEI was *r* = 0.285 (*N* = 240; *p* < 0.001), while for females it was *r* = 0.312 (*N* = 234, *p* < 0.001).

A number of regressions were then run. Table 3 shows a simple regression with SEI as the criterion variable. The predictor variables were three demographic factors, IQ and three self-ratings. It was significant, accounting for 30.9% of the variance. The most powerful correlate was SEEQ, then IQ, followed by participant sex. No other variables were significant.

Two variables were then computed. Self-rated attractiveness and health were combined into a self-rating score. Also, religiousness and belief in life after death were combined into a belief score. Then, a stepwise regression was computed which was significant (F(6,452) = 19.94, *p* < 0.001, Adj *R*^2^ = 17.3). The first step was sex (F(1,456) = 24.82, *p* < 0.001, *R*^2^ = 0.05), then self-rating of attractiveness and health combined (F(2,455) = 23.08, *p* < 0.001, *R*^2^ = 0.09), then religion and beliefs in life after death combined (F(3,454) = 15.43, *p* < 0.001, *R*^2^ = 0.09), then CTs (F(4,453) = 14.06, *p* < 0.001, *R*^2^ = 0.11) and finally IQ (F(5,452) = 19.73, *p* < 0.001, *R*^2^ = 0.18). These results suggest that it is predominantly sex, self-ratings and IQ that relate to SEI.

## 4. Discussion

This study explored various correlates of SEI. The study confirmed the previous, almost universal findings concerning sex differences in SEI, namely that males give significantly higher self-estimates than females. These studies showed, as before, that people tend to believe they are about 0.5 of an SD above the norm, though this was less pronounced in the second study. Previous studies have shown small age and education effects, in which younger people and better educated people tend to give a higher SEI. These effects were less evident in this sample, no doubt because of the relative homogeneity of these samples concerning these two variables.

The second study also confirmed that data suggest IQ and SEI are significantly positively correlated at *r* = 0.30. There has long been a question of whether males tend to over-rate their scores or females under-rate their scores. The second study showed some evidence that females are more accurate estimators of their own ability than males, though the difference was not large. Previous studies have shown that those who have done IQ tests and believe in their validity tend to give higher SEI estimates (Furnham 2001). This could be because of a number of factors: brighter people are more likely to take tests (showing they are brighter), the feedback they get makes them more self-confident, doing a test makes them clearer about the concept of IQ.

The focus of this study was on two additional types of correlates of SEI. The first was self-beliefs, operationalised as self-ratings of attractiveness and health as well as emotional intelligence. The idea was that most self-ratings are positively correlated into an overall self-concept and self-esteem measure, with people feeling more or less positive about themselves. It was therefore predicted and tested whether these ratings were correlates of SEI. Both studies showed this to be the case, with correlations in the first study ranging between 0.33 and 0.44 and, in the second, study ranging from 0.21 to 0.22 for self-ratings of attractiveness and health. Whilst the mechanism or process of this is unclear, it supports the vast self-concept literature, now extended to the SEI literature. The question remains as to what self-ratings of specific skills (i.e., negotiation, presentation) or other attributes (i.e., strengths like courage, kindness) are also positively associated with SEI and why.

Indeed, Judge et al. (1998) proposed the concept of core self-evaluations (CSE) which were defined as a broad personality trait reflecting the most general and fundamental beliefs individuals hold about themselves. The CSE comprises four components referring to individual differences in (a) self-esteem or perception of one’s worth, value, and importance, (b) generalized self-efficacy or one’s typical level of confidence in the likelihood of performing well, (c) locus of control or one’s perceived degree of control over life events and situations, and (d) neuroticism/emotional stability or one’s tendency to experience negative affects, increased levels of worry, and pessimistic beliefs. A large meta-analytic study (Judge and Bono 2001) confirmed that most variance among these components can be explained by a general factor, which was more accurate than the four components in predicting external criteria. Earlier Chamorro-Premuzic et al. (2008) showed that SEI was correlated *r* = 0.21 (N = 388) with CSE.

The study also tested the idea that (scientific) skepticism, as operationalised by being religious, believing in alternative medicine, life after death, and CTs, is related to SEI. These were mostly all significantly and logically related. In both studies, we examined the relationship between religiousness and belief in alternative medicine and SEI. Belief in CAM was negatively correlated with SEI in both studies but only significantly in Study 2. Surprisingly, in Study 1, a positive significant correlation was found between religiousness and SEI, but there was no significant relationship between religiousness and SEI in Study 2. However, in Study 2 we did establish that SEI was correlated with not believing in the afterlife (a major cornerstone of all religions) and a lower belief in CTs. Various studies have shown that more intelligent people tend to be less religious, and less likely to be believers in CTs and alternative medicine. This study showed this to be equally true of SEI.

Both studies also considered the relative power of different kind of variables to explain SEI. We examined demographic variables (sex, age, education), self-ratings (attractiveness, EQ and health), non/anti scientific beliefs (CAM, CT and religion), as well as test-derived IQ. The results showed that while self-ratings and non-science beliefs contribute significant variance to the explanation of SEI, it is predominantly sex and psychometric IQ that accounts for most of the variance.

With only one exception in this now extensive literature of over 50 studies dating back 50 years, males have given significantly higher self-estimates than females. This is not justified by the IQ test literature, which suggests minimal differences (Furnham 2001). However, this male hubris effect is found in a very wide range of self-assessments of ability and attractiveness, suggesting that intelligence may not be particularly special (Voges et al. 2019).

## Figures and Tables

**Table 1:** Pearson product–moment correlations of variables in the first study.

	Variables	1	2	3	4	5	6	7	8	9
1	Gender									
2	Age	0.249 ***								
3	Edu	0.044	0.152 **							
4	SR of Physical Attract	−0.116 **	−0.046	0.041						
5	SR of Physical Health	−0.078	0.022	0.069	0.394 ***					
6	Religious	0.006	0.076	0.070	0.204 ***	0.143 **				
7	Optimistic	0.058	−0.057	0.003	0.356 ***	0.286 ***	0.184 ***			
8	Belief in CAM	0.196 ***	0.185 ***	−0.055	0.085	0.073	0.246 ***	0.159 ***		
9	SEI	−0.093 *	0.051	0.096 *	0.441 ***	0.326 ***	0.193 ***	0.221 ***	−0.004	
10	SEEQ	−0.092 *	0.079	0.029	0.370 ***	0.296 ***	0.113 *	0.288 ***	0.077	0.390 ***

Note: * *p* < 0.05, ** *p* < 0.01, *** *p* < 0.001.

**Table 2:** Pearson product-moment correlations of variables in the second study.

	Variables	1	2	3	4	5	6	7	8	9	10	11
1.	Sex											
2.	Age	−0.003										
3.	Edu	0.015	−00.48									
4.	SR Attract	−0.130 **	0.008	−0.004								
5.	SR Health	−0.129 **	0.118 **	0.017	0.525 ***							
6.	Relig	0.041	0.086	0.017	0.005	−0.011						
7.	Optim	0.001	0.032	0.012	0.284 ***	0.243 ***	0.167 ***					
8.	CAM	0.191 ***	0.170 ***	−0.056	0.108 *	0.078	0.279 ***	0.317 ***				
9.	CT	0.068	−0.085	0.159 **	0.058	−058	0.142 **	0.055	0.341 ***			
10	SEI	−0.218 ***	0.084	0.071	0.222 ***	0.205 ***	−0.044	0.006	−0.130 **	−0.143 **		
11	SEEQ	0.022	0.004	0.027	0.229 ***	0.209 ***	0.118 **	0.185 ***	0.029	0.032	0.417 ***	
12	IQ	−0.019	0.087	0.042	0.047	0.033	−0.107 *	−0.094 *	−0.079	−0.142 **	0.295 ***	0.059

Note: * *p* < 0.05, ** *p* < 0.01, *** *p* < 0.001.

**Table 3:** Regression.

Variables	SE-IQ		
	*F*(7,463) = 29.583 ***, *R*^2^ = 0.31		
	*B*	*β*	*t*
Gender	−5.90	−0.21	−5.35 ***
Age	0.07	0.06	1.52
Education	0.66	0.06	1.47
IQ	2.07	0.25	6.41 ***
SE-EQ	0.40	0.38	9.39 ***
SE-ATTRACT	0.05	0.07	1.54
SE-HEALTH	0.03	0.05	1.11

Note: *** *p* < 0.001.
